# Statement of Concern

**DOI:** 10.1097/MD.0000000000021299

**Published:** 2020-07-17

*Medicine* is publishing a statement of concern regarding two *Medicine* articles.

The journal was alerted to concerns alleging improper statistical analysis in published article “Effects of high-intensity interval and moderate-intensity continuous aerobic exercise on diabetic obese patients with nonalcoholic fatty liver disease”^[1]^ which appeared in Volume 99, Issue 10 of *Medicine*. After investigation, it was found that the analysis performed was inappropriate and as a result, the data must be re-analyzed and corrected appropriately. It was also alleged that in published article “A randomized controlled trial on the effectiveness of 8-week high-intensity interval exercise on intrahepatic triglycerides, visceral lipids, and health-related quality of life in diabetic obese patients with nonalcoholic fatty liver disease”^[2]^ appearing in Volume 98, Issue 12 of *Medicine*, between-group tests are reported using the final measure. However it does not seem to be correctly calculated. It was also determined through investigation that the ‘interaction effect’ of exercise on primary (and subsequent outcome measures) cannot be statistically interrogated in.^[2]^

Additionally, a concern was raised regarding potential duplication of results in^[1]^ that were reported in^[2]^ and “Effect of Moderate-Intensity Aerobic Exercise on Hepatic Fat Content and Visceral Lipids in Hepatic Patients with Diabesity: A Single-Blinded Randomised Controlled Trial”^[3]^ which appeared in Volume 2020 of *Evidence-Based Complementary and Alternative Medicine*. After investigation, it appears duplication did not occur as^[1]^ presents an experimental arm (MICT), although authors appear to have failed to include deviations to experimental arms in the clinical trial registration.

The original Letter to the Editor documenting the concerns has been published on our Author Correspondence blog. Authors and their institutions have been contacted to address the concerns, re-analyze data, and ensure all clinical trial registration information is accurate.
